# Exciton-acoustic phonon coupling revealed by resonant excitation of single perovskite nanocrystals

**DOI:** 10.1038/s41467-021-22486-5

**Published:** 2021-04-13

**Authors:** Yan Lv, Chunyang Yin, Chunfeng Zhang, Xiaoyong Wang, Zhi-Gang Yu, Min Xiao

**Affiliations:** 1 National Laboratory of Solid State Microstructures, School of Physics, and Collaborative Innovation Center of Advanced Microstructures, Nanjing University, Nanjing, 210093 China; 2 ISP/Applied Sciences Laboratory, Washington State University, Spokane, WA 99210 USA; 3 Department of Physics and Astronomy, Washington State University, Pullman, WA 99164 USA; 4 Department of Physics, University of Arkansas, Fayetteville, AR 72701 USA

**Keywords:** Fluorescence spectroscopy, Single photons and quantum effects

## Abstract

Single perovskite nanocrystals have attracted great research attention very recently due to their potential quantum-information applications, which critically depend on the development of powerful optical techniques to resolve delicate exciton photophysics. Here we have realized resonant and near-resonant excitations of single perovskite CsPbI_3_ nanocrystals, with the scattered laser light contributing to only ~10% of the total collected signals. This allows us to estimate an ultranarrow photoluminescence excitation linewidth of ~11.32 µeV for the emission state of a single CsPbI_3_ nanocrystal, corresponding to an exciton dephasing time of ~116.29 ps. Meanwhile, size-quantized acoustic phonons can be resolved from a single CsPbI_3_ nanocrystal, whose coupling with the exciton is proposed to arise from the piezoelectric potential. The ability to collect resonance fluorescence from single CsPbI_3_ nanocrystals, with the subsequent revelation of exciton-acoustic phonon coupling, has marked a critical step towards their steady advancement into superior quantum-light sources.

## Abstract (web summary)

In order to develop perovskite nanocrystals as a single-photon source, there is a need to understand the complex exciton photo-physics. Here, the authors employ resonant and near-resonant excitation technique to study single CsPbI_3_ nanocrystal that allows them to probe the continuous and size-quantised acoustic-phonon modes.

## Introduction

Semiconductor perovskite nanocrystals (NCs) have attracted great research attention in the single-particle optical studies since their first successful synthesis in 2015 (ref. ^1^), with the subsequent observations of single-photon emission^2,3^, suppressed photoluminescence (PL) blinking and spectral diffusion^4^, and stable exciton fine structures^5,6^. By means of photon-correlation Fourier spectroscopy, the PL linewidth was measured to be ~17.0 μeV for the emission-state excitons of single CsPbBr_3_ NCs^7^, while the exciton dephasing time of the absorption state could be longer than ~10 ps in single CsPbI_3_ NCs based on the quantum interference measurement^8^. These coherent optical properties, which are rarely achievable in traditional colloidal NCs despite several decades of active pursuits, promise great potential of single perovskite NCs in quantum-information applications. In the aforementioned pioneering works^7,8^, a single perovskite NC was first excited into the absorption state with the coherent information being extracted next from the emission state, which would cause not only linewidth broadening to reduce the exciton coherence^9,10^ but also timing jitter in the photon generation and emission events^11^. To circumvent such undesired situations in the coherent optical studies of single perovskite NCs, it is imperative to realize resonant excitation of the emission state. This is analogous to the historical development of single epitaxial quantum dots (QDs), whose routine demonstrations of Mollow-triplet spectra^12^ and indistinguishable single photons^13^ are critically dependent on the ability to collect the resonance fluorescence^14^.

Even under resonant excitation of a single epitaxial QD, it is still possible for the emission state to be disturbed by environmental spin and charge fluctuations^15,16^, as well as lattice vibrations of the acoustic-phonon modes^16–18^. Specifically, the exciton-acoustic phonon coupling could induce a broad sideband around the emission state, which fundamentally determines the upper limits that can be achieved for the lifetime of exciton coherence and the degree of photon indistinguishability^19,20^. The soft ionic lattice of semiconductor perovskites is featured with a strong anharmonicity^21–23^ to bring about low-energy and short-lived acoustic phonon modes^23–27^, with the accompanied low thermal conductivity^28,29^ and strong acoustic-optical phonon up-conversion^30^. These acoustic phonons can significantly influence the carrier transport and relaxation dynamics of semiconductor perovskites at the cryogenic temperatures. In contrast to the optical phonons that have been widely studied in the literature^31,32^, the acoustic phonons are yet to be experimentally detected in single perovskite NCs, let alone their possible influences on the exciton photophysical properties.

By adopting an orthogonal polarization geometry for laser excitation and PL collection, we show here that resonance fluorescence can be collected from single perovskite CsPbI_3_ NCs at the cryogenic temperature, with the residual contribution of scattered laser light being as low as ~10%. This allows us to resolve an ultranarrow PL excitation linewidth of ~11.32 μeV for the emission state of a single CsPbI_3_ NC, corresponding to a dephasing time of ~116.29 ps for the band-edge excitons. We further demonstrate that a single CsPbI_3_ NC can be efficiently excited when the laser energy is tuned across hundreds of μeV both above and below its emission state, which unambiguously confirms the participation of continuous acoustic phonons in the exciton generation processes. Moreover, a size-quantized acoustic-phonon mode is revealed under both near-resonant and resonant excitations of single CsPbI_3_ NCs, whose energy changes from ~150 to ~180 μeV with increase of the exciton’s emission energy from ~1.70 to ~1.73 eV due to the reduction of NC size.

## Results

### Chemical synthesis and optical setup

The perovskite CsPbI_3_ NCs are synthesized according to a standard hot-injection method^5^ (see Supplementary Methods in the Supplementary Information) with a cubic edge length of ~9.31 ± 0.68 nm (see the transmission electron microscopy image in Supplementary Fig. 1). Meanwhile, their long-term stability is obtained by the addition of tri-octylphosphine ligands in the post-synthesis treatment^33^. One drop of the diluted NC solution is spin-coated onto a fused silica substrate, which is then attached to the cold finger of a helium-free cryostat for the optical studies of single CsPbI_3_ NCs. The experimental setup is schematically shown in Supplementary Fig. 2 (see Supplementary Methods in the Supplementary Information), where a He-Ne laser (~1.96 eV) and a tunable diode laser (~1.687–1.739 eV) both operated at the continuous-wave mode are employed for the above-bandgap and resonant/near-resonant excitations, respectively. For the purpose of observing resonance fluorescence from a single CsPbI_3_ NC, two Glan-Thompson polarizers with orthogonal transmission axes are inserted into the laser excitation and PL collection paths, respectively, along with a quarter-wave plate to correct the birefringence effect caused by the relevant optical components^20,34^ (see Supplementary Fig. 3). Unless otherwise specified in the text, all the optical measurements are performed at the cryogenic temperature of 3 K, and the laser excitation power is normally set at ~100–500 nW so that the PL intensity of a single CsPbI_3_ NC is not saturated to minimize the possibility of generating multiple excitons (see Supplementary Fig. 4). Within this range of laser excitation power, all the single CsPbI_3_ NCs studied in our experiment possess a very good chemical stability without suffering from the photo-bleaching effect during the measurement time as long as 15 min.

### Resonance fluorescence of single CsPbI_3_ NCs

**Fig. 1:**
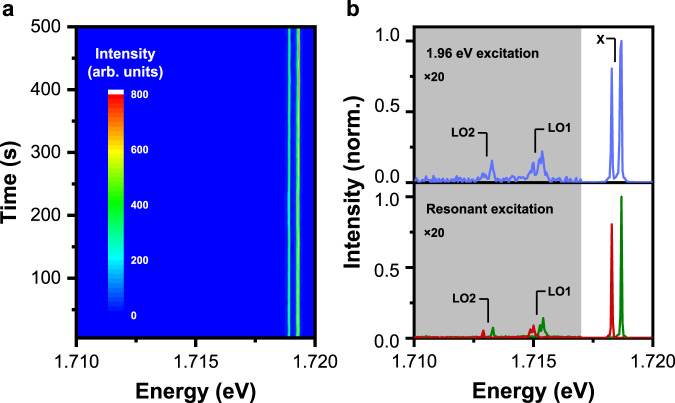
Fundamental optical properties of a single CsPbI_3_ NC. **a** Time-dependent PL spectral image constructed from 100 PL spectra each acquired with an integration time of 5 s under the 1.96 eV laser excitation. **b** (top panel) PL spectrum plotted by summing over the 100 PL spectra used in **a** to construct the image. (bottom panel) Two PL spectra measured with the laser excitation energies being resonant with the higher- and lower-energy PL peaks, respectively, both using an integration time of 1 s. In **b**, PL intensities in the shaded areas are magnified by 20 times, while X and LO1/LO2 denote the single-exciton doublet peaks and the two longitudinal-optical phonon modes, respectively.

In Fig. 1a, we plot the time-dependent PL spectral image of a representative single CsPbI_3_ NC excited at 1.96 eV, where the doublet PL peaks from the single-exciton fine structure^5,6^ can be clearly resolved with orthogonal linear polarizations. By summing over the 100 PL spectra each acquired with an integration time of 5 s for the construction of this image, we show in Fig. 1b (top panel) that two sets of doublet PL peaks from longitudinal-optical (LO) phonons emerge from the background level in addition to the single-exciton one. These two LO phonon modes should arise from the Pb-I-Pb bending motions with the respective energies of ~3.29 and ~5.29 meV, as revealed previously from the Raman and neutron scattering measurements^25,28^. In the bottom panel of Fig. 1b, by tuning the diode laser energy to either of the doublet peak positions, we further plot two PL spectra each obtained with an integration time of 1 s. Compared with the 1.96 eV excitation at the same laser power, PL intensities of the single-exciton doublet peaks are both enhanced under resonant excitations by almost two orders of magnitude, and the two LO phonon modes can be more easily resolved owing to the significantly-reduced background noise. To estimate the residual contribution of scattered laser light to the resonance fluorescence of the single-exciton doublet peaks (Fig. 1b, bottom panel), we select within the laser spot one blank area and another area with a single CsPbI_3_ NC whose optical signals can be simultaneously collected by the CCD camera for the comparison purpose.

**Fig. 2:**
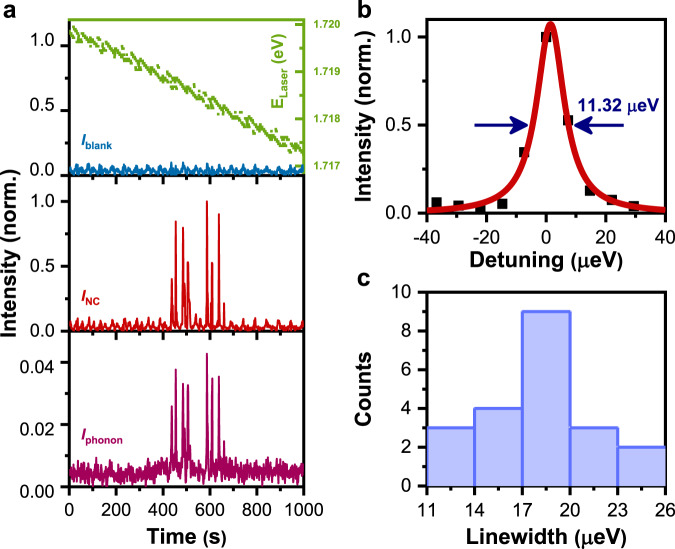
Resonance fluorescence from a single CsPbI_3_ NC. **a** (top panel) Time-dependent energy and intensity of the moving laser peak scanned from ~1.720–1.717 eV on the blank sample area. (middle panel) The same intensity measurement on the single-NC sample area. (bottom panel) Integrated PL intensity of the two LO phonon modes plotted as a function of the measurement time. **b** PL intensity of the lower-energy peak plotted as a function of the detuned laser energy and fitted with a linewidth of ~11.32 µeV for this single CsPbI_3_ NC. **c** Statistical histogram showing the linewidth distribution of the PL excitation spectra measured for the emission states of 21 single CsPbI_3_ NCs.

As shown in Fig. 2a for the blank area (top panel), when the laser energy is scanned over time from ~1.720–1.717 eV with a step resolution of ~7.35 μeV/s, the monitored intensity of the moving laser peak on the CCD camara shows small fluctuations around a constant value. For the single-NC area in the middle panel of Fig. 2a, there appear two batches of intensity spikes during ~430–510 and ~580–660 s when the laser energy is being tuned to the doublet-peak positions with the higher and lower energies, respectively. This indicates that resonance fluorescence of a single CsPbI_3_ NC has been successfully collected during these two time periods, which is supported by the similar trend observed in the bottom panel of Fig. 2a from the integrated PL intensity of the two LO phonon peaks. Owing to the mode-hopping effect of the diode laser energy (Fig. 2a, top panel), each of the doublet peaks can be excited multiple times during the scanning, yielding several intensity spikes instead of a single one (Fig. 2a, middle and bottom panels). This mode-hopping effect and the accompanied variation in the laser power, as reflected in the fluctuating background signals in the top and middle panels of Fig. 2a, give rise to the intensity differences among these spikes. By comparing the maximum intensity of these spikes with that of the background signal (Fig. 2a, middle panel), we can estimate that the contribution of scattered laser light to each of the resonantly-excited PL spectra in Fig. 1b (bottom panel) is as low as ~10%.

On the same single CsPbI_3_ NC, we next tune the laser energy across a very small range around either of the doublet peaks, so that their corresponding changes of the PL intensities can be smoothly measured without suffering from the mode-hopping effect. As shown in Fig. 2b for the lower-energy peak, the PL excitation spectrum can be fitted by a Lorentzian function with a linewidth of ~11.32 μeV. This kind of PL excitation measurement has been performed for a total of 21 single CsPbI_3_ NCs in our experiment, and an average linewidth of ~17.62 μeV can be calculated from the distribution histogram shown in Fig. 2c. For the single CsPbI_3_ NC with the PL excitation linewidth of ~11.32 μeV (Fig. 2b), it can be judged from the time-dependent PL spectral image in Fig. 1a that there exists no spectral diffusion within our system resolution of ~100 μeV. In contrast, for single CsPbI_3_ NCs with broader linewidths such as ~17.40 μeV (Supplementary Fig. 5a), the single-exciton doublet peaks suffer obviously from the spectral diffusion effect with the occasional transition to a charged-exciton peak (Supplementary Fig. 5b). The above observations suggest that charge fluctuations could be one likely cause to broaden the PL excitation linewidth of a single CsPbI_3_ NC, which might be also influenced by the exciton-acoustic phonon coupling effect as discussed below.

### Exciton-acoustic phonon coupling

**Fig. 3:**
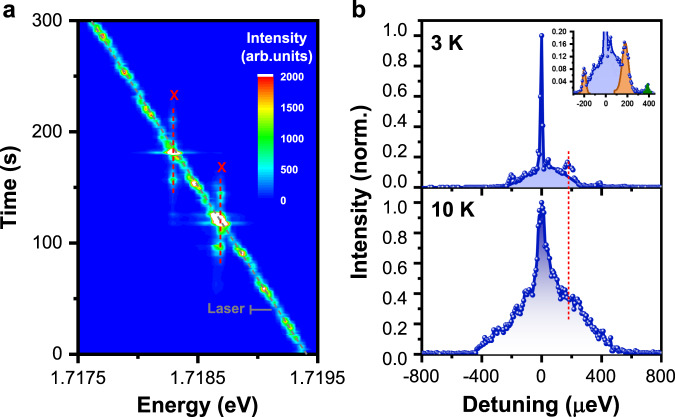
Continuous acoustic phonons in a single CsPbI_3_ NC. **a** 2D image showing intensity variations of the doublet PL peaks with the laser scanning energies from ~1.7175–1.7195 eV. Here X denotes the energy position for either of the doublet PL peaks. **b** (top panel) PL intensity of the lower-energy peak plotted as a function of the detuned laser energy at 3 K. Inset: Amplified view of the low-signal region. (bottom panel) PL intensity of the lower-energy peak plotted as a function of the detuned laser energy at 10 K. The dotted line marks the energy position where the phonon-assisted excitation efficiencies of ~16.6 and ~37.1% can be achieved at 3 and 10 K, respectively, as compared to that of the resonant excitation at 0 µeV. See Supplementary Fig. 6 and related discussions in the Supplementary Information for theoretical fits of the PL excitation spectra measured at 3 and 10 K.

In Fig. 3a, we construct a 2D image for a single CsPbI_3_ NC to show in detail how the PL intensities of its doublet peaks evolve within a larger range of laser scanning energies from ~1.7175–1.7195 eV. In addition to the resonant enhancement, each of the doublet peaks can still be effectively excited when the laser energy is detuned to its blue or red side by hundreds of µeV. As can be seen in the top panel of Fig. 3b with a variation of the detuned laser energy, PL intensity of the lower-energy peak rises from −250 μeV to reach a maximum at 0 μeV and then decays to the background level at 350 μeV. This broad PL excitation spectrum is associated with an asymmetric lineshape, showing that an excitation efficiency larger than ~16.6% can be achieved by the blue-detuned laser as compared to that of the resonant excitation. When the sample temperature is increased to 10 K (Fig. 3b, bottom panel), the lower-energy peak of a single CsPbI_3_ NC can now be excited within a larger energy range from −450 to 600 μeV, with the excitation efficiency being elevated to more than ~37.1% for the blue-detuned laser. The above results strongly suggest the involvement of acoustic phonons with continuous energies in the exciton down- and up-conversion processes under the blue- and red-detuned laser excitations, respectively, as reported previously from similar optical studies of single epitaxial InGaAs QDs^10^.

**Fig. 4:**
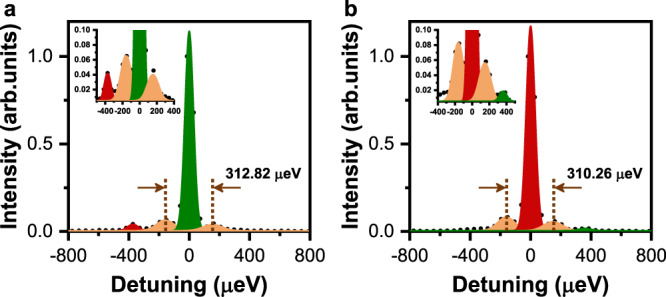
Size-quantized acoustic phonons in a single CsPbI_3_ NC. **a** Resonantly-excited higher-energy peak (green), the associated side peaks from size-quantized acoustic photons (orange), and the down-converted lower-energy peak (red). Inset: Amplified view of the low-signal region. See Supplementary Fig. 7 and related discussions in the Supplementary Information for theoretical fit of this resonantly-excited PL spectrum. **b** Resonantly-excited lower-energy peak (red), the associated side peaks from size-quantized acoustic photons (orange), and the up-converted higher-energy peak (green). Inset: Amplified view of the low-signal region.

As shown in the inset of Fig. 3b (top panel), two side peaks can be additionally resolved at about −190 and 190 μeV from the PL excitation spectrum that has been replotted for an amplified view of the low-signal region. In fact, these two side peaks also appear in the PL spectral image of Fig. 3a when either of the doublet peaks is being resonantly excited. In Fig. 4a and b, we further plot two resonantly-excited PL spectra for the higher- and lower-energy emission states of another single CsPbI_3_ NC, respectively. By fitting each PL spectrum with multiple Gaussian functions, the two side peaks are estimated to be ~155 μeV below and above the central peak with an intensity ratio of ~1.50. Such set of down- and up-converted side peaks has been universally observed in the 24 single CsPbI_3_ NCs studied in our experiment, from which an average intensity ratio of ~1.47 ± 0.23 can be obtained. Meanwhile, when the emission energy of the central PL peak increases from ~1.70–1.73 eV in different single CsPbI_3_ NCs, each side peak is detuned away from it with an increasing energy separation from ~150–180 μeV (Supplementary Fig. 8). Since this energy separation is independent of the laser excitation power (Supplementary Fig. 9), the Mollow-triplet origin^12^ can be safely excluded. Nor could they be attributed to the exciton fine structures and LO phonon modes, which have been well resolved in our experiment. Then the two side peaks should correspond to a size-quantized acoustic-phonon mode arising from the spatial confinement of lattice vibrations^35^.

Although these size-quantized acoustic phonons can be easily detected under resonant excitation, they only show up in the PL spectra of a limited number of single CsPbI_3_ NCs excited above the bandgap at 1.96 eV (Supplementary Fig. 10) due to the poor signal-to-noise ratio mentioned earlier in the text. In Supplementary Fig. 11, we plot the PL spectra of two such single CsPbI_3_ NCs excited at 1.96 eV with the emission energies of ~1.7332 and ~1.7464 eV, respectively, and the associated energies of their size-quantized acoustic phonons are estimated to be ~210 and ~260 μeV, respectively. The above observations thus further confirm the increasing trend of the size-quantized acoustic-phonon energies with the increasing NC emission energies, which deserves intensive future studies with different CsPbI_3_ NC samples and tunable diode lasers other than those employed in the current experiment.

The influences of acoustic phonons on the exciton emission linewidths and relaxation dynamics were previously reported in traditional colloidal NCs^36–43^, however, this is the first time that they have been detected directly from the optical studies of single perovskite NCs. In general, the exciton-acoustic phonon coupling can originate from either the short-range deformational potential, or the long-range piezoelectric potential if the material lacks an inversion symmetry^35^. In the former case, the coupling is due mainly to the longitudinal acoustic (LA) phonons when the conduction and valence bands are approximately isotropic as in the perovskite CsPbI_3_ material. Whereas in the latter case, both LA and transverse acoustic (TA) phonons can be involved in the exciton-acoustic phonon coupling. In perovskite materials, the piezoelectric coupling between excitons and TA phonons is particularly strong (see theoretical calculations in the Supplementary Information). For a small phonon wave vector *q*, the deformational and piezoelectric coupling strengths are $V_d(q)\sim q^{1/2}$ and $V_p(q)\sim q^{ - 1/2}$, respectively, implying that the piezoelectric coupling would reach a maximum for the smallest wave vector allowed by a finite-sized NC. The small-*q* acoustic phonons, with their wavelengths (approximately the NC size) being much longer than the lattice constant, can be described by the elastic continuum theory, and their wave velocity and polarization along a given direction can be obtained by solving the Christoffel equation^44^.

In particular, for the acoustic wave propagating along the symmetry axes such as [100], there exist one LA mode with the velocity $v_l = (C_{11}/\rho )^{1/2}$ and two TA modes with the velocity $v_t = (C_{44}/\rho )^{1/2}$, where *C*_11_ is the axial compression modulus, *C*_44_ is the shear modulus, and *ρ* is the mass density. Using the theoretical values of *C*_11_ = 34.23 GPa, *C*_44_ = 3.24 GPa and *ρ* = 5.39 g/cm^3^ reported in the literature for perovskite materials^45,46^, we can obtain the velocities of *ν*_*l*_ = 2.52 × 10^3^ and *ν*_*t*_ = 0.78 × 10^3^ m/s, respectively. Since the smallest wave vector along the symmetry axis in a single CsPbI_3_ NC is $q = \pi /L$ with *L* being the edge length, the corresponding energies of the LA and TA modes are $\hbar \omega _l = \hbar v_lq$ = 560 μeV and $\hbar \omega _t = \hbar v_tq$ = 172 μeV, respectively. Thus, the energy range of ~150–180 μeV (Supplementary Fig. 8) obtained for the size-quantized acoustic phonons should be related to the TA modes, which couple strongly with the excitons via the piezoelectric potential (see theoretical calculations of the Supplementary Information). On the other hand, the coupling of excitons with continuous acoustic phonons, as observed here in single CsPbI_3_ NCs (Fig. 3b) and previously in many other inorganic nanostructures^47^, could proceed through weaker piezoelectric and deformational potentials that couple to LA phonons.

### Influence of acoustic phonons on exciton transitions

When the higher-energy peak of a single CsPbI_3_ NC is being resonantly excited, it can be seen from the inset of Fig. 4a that the lower-energy peak ~370 µeV below also shows up in addition to the size-quantized acoustic-phonon modes. When the laser energy is off this resonant position by only several μeV, PL intensity of the lower-energy peak is reduced almost completely to the background level. This suggests that the lower-energy state is not populated directly by the laser excitation, but instead via exciton relaxation from the higher-energy state by emitting acoustic phonons. However, this exciton relaxation process is extremely inefficient, as reflected by the intensity ratio of ~0.040 between the lower- and higher-energy PL peaks. Similarly shown in the inset of Fig. 4b, the higher-energy peak can also be detected when the lower-energy peak is being resonantly excited, albeit with a much smaller ratio of ~0.015 between their PL intensities in this exciton up-conversion process. Based on the fact that the two emission states have an energy-level splitting of ~370 μeV, which is obviously larger than the maximum acoustic phonon energy of ~350 μeV detected in a single CsPbI_3_ NC, the above observations can be understood via a higher-order multiple phonon process with the piezoelectric coupling. These two emission states are associated with bright excitons of a single CsPbI_3_ NC^5^, and it was revealed very recently from a magneto-optical study that they are several meV higher in energy than the dark-exciton state^48^. This makes it even more difficult for the bright excitons to relax into the dark state, which naturally explains why the emission states of a single CsPbI_3_ NC are highly luminescent, without seeking the Rashba effect to place the dark state in the highest-energy position^6^ or a two-optical phonon Raman scattering process for the mixing of bright and dark states^31^. It should be noted that the exciton transition process between the bright states could be thermally enhanced due to the availability of more acoustic phonons, as is shown in Supplementary Fig. 12 from the resonantly-excited PL spectra measured for a single CsPbI_3_ NC at different temperatures.

## Discussion

To summarize, we have realized resonant and near-resonant excitations of single perovskite CsPbI_3_ NCs, with the scattered laser light accounting for as low as ~10% of the total collected signals. Further reduction of the residual laser light is possible when the excitation beam is incident on the sample surface with a tilted angle, instead of passing through the objective confocally in our experiment. However, the current optical setup has already allowed us to probe the continuous and size-quantized acoustic-phonon modes with a high signal-to-noise ratio, whose energies are distributed within the ranges of 0–350 and 150–180 μeV, respectively. These acoustic phonons are indispensable in assisting the detuned laser excitation of a single CsPbI_3_ NC, allowing us to resolve an ultranarrow PL excitation linewidth of ~11.32 μeV that can be regarded as an upper limit for the intrinsic emission-state PL linewidth. The transformed exciton dephasing time of ~116.29 ps is almost one order of magnitude shorter than the exciton radiative lifetime of ~1 ns^5^, the latter of which can be surely accelerated in future works by coupling a single CsPbI_3_ NC to a plasmonic nanocavity^49^ or a dielectric waveguide^50^ for the generation of indistinguishable single photons. Our results indicate that, so long as the acoustic-phonon energies are smaller than the fine energy-level splittings, thermal mixing among the bright and dark states is greatly minimized to ensure a long exciton dephasing time. Overall, the ability to collect resonance fluorescence and the revelation of acoustic-phonon modes as well as their role in the exciton dephasing process represent a critical step in the development of single CsPbI_3_ NCs as a superior single-photon source for quantum-information applications.

## Supplementary information

Supplementary Information

## Data Availability

The data supporting the findings of this study are available from the corresponding authors upon request.
