# Supplementary material for: Exciton-acoustic phonon coupling revealed by resonant excitation of single perovskite nanocrystals
Source: Nat Commun. 2021 Apr 13;12:2192. doi: 10.1038/s41467-021-22486-5 (PMC8044187; doi:10.1038/s41467-021-22486-5)
Supplement: Supplementary file 1 — Supplementary Information [file 41467_2021_22486_MOESM1_ESM.pdf]

Supplementary Information

Exciton-Acoustic Phonon Coupling Revealed by Resonant Excitation of Single Perovskite Nanocrystals

Yan Lv[1†], Chunyang Yin[1†], Chunfeng Zhang[1], Xiaoyong Wang[1*], Zhi-Gang Yu[2*], and Min Xiao[1,3*]

[1]*National Laboratory of Solid State Microstructures, School of Physics, and Collaborative Innovation Center of Advanced Microstructures, Nanjing University, Nanjing 210093, China*

[2]*ISP/Applied Sciences Laboratory, Washington State University, Spokane, Washington 99210; Department of Physics and Astronomy, Washington State University, Pullman, Washington 99164, USA*

[3]*Department of Physics, University of Arkansas, Fayetteville, Arkansas 72701, USA*

[*]Correspondence to X.W. (wxiaoyong@nju.edu.cn), Z.Y. (zhi-gang.yu@wsu.edu) or M.X. (mxiao@uark.edu)

[†]These authors contributed equally to this work

Supplementary Methods

Chemical synthesis. To prepare the Cs-oleate precursor, Cs_2CO_3 (0.54 g), oleic acid (OA, 1.7 mL) and octadecene (ODE, 20 mL) were loaded into a 100 mL three-neck flask and dried under vacuum for 1 h at 120 ℃. The solution was then heated to 150 ℃ under N_2 until it became clear. For the synthesis of the $CsPbI_3$ NCs, PbI_2 (0.174 g), ODE (10 mL), OA (0.5 mL) and oleylamine (OLA, 0.5 mL) were loaded into a 50 mL three-neck flask and dried under vacuum for 1 h at 120 ℃. The temperature of this mixture was raised to 160 ℃ subsequently, into which the preheated Cs-oleate solution (0.8 mL) was swiftly injected. After 5 s, the reaction mixture was transferred to the ice-water bath and 1 mL of tri-octylphosphine was injected at the temperature of 80 ℃. To purify the $CsPbI_3$ NCs, the resulting solution was centrifuged for 5 min at 4000 rpm and the suspension was further centrifuged for 10 min at 10000 rpm. Finally, the precipitate was dispersed in hexane and stored in the glove box.

Optical measurements. One drop of the diluted NC solution was spin-coated onto a fused silica substrate, where only several bright spots could be detected from the confocal scanning PL image measured for a sample area of 10 μm × 10 μm. Such a bright spot corresponds to the optical emission from a single $CsPbI_3$ NC, as confirmed previously from the photon anti-bunching measurement in ref. 5 of the main text. The sample substrate was attached to the cold finger of a helium-free cryostat, while a He-Ne laser and a tunable diode laser both operated at the continuous-wave mode were employed for the above-bandgap and resonant/near-resonant excitations of a single $CsPbI_3$ NC, respectively. The laser beam was focused onto the sample substrate by a dry objective with a numerical aperture of 0.82 and the optical signal collected by the same objective was sent to a spectrometer (0.75 m, 1200 g/mm grating) and a charge-coupled device (CCD) camera for the spectral measurement with a resolution of ~100 μeV. For the purpose of observing resonance/near-resonance fluorescence from a single $CsPbI_3$ NC

excited by the tunable diode laser, two Glan-Thompson polarizers with orthogonal transmission axes were inserted into the laser excitation and PL collection paths, respectively. A quarter-wave plate was mounted to a stage with a rotating precision of 0.01 degree to correct the birefringence effect caused by the relevant optical components. To further suppress the scattered laser light, the entrance slit of the spectrometer was closed to ~120 µm for the spatial filtering and an extinction ratio exceeding 10^5 could be obtained at a typical laser wavelength of 725 nm. As for the above-bandgap excitation, a band-pass optical filter was used to separate the PL signal of a single $CsPbI_3$ NC and the scattered light from the He-Ne laser. Unless otherwise specified in the text, all the optical measurements were performed at the cryogenic temperature of 3 K, and the laser excitation power was normally set at ~100-500 nW so that the PL intensity of a single $CsPbI_3$ NC was not saturated to minimize the possibility of generating multiple excitons.

Theoretical Calculations

Modelling details. The acoustic waves in a solid satisfy the following differential equation [ref. S1],

$$\frac{\partial}{\partial x_i}\left(c_{ijkl}\frac{\partial u_k}{\partial x_l}\right) = \rho\frac{\partial^2 u_i}{\partial t^2},$$

where $u_{i,k}$ is the lattice displacement, c_{ijkl} is the stiffness tensor, and ρ is the mass density. For a plane wave, the lattice displacement can be written as

$$u_k \propto P_k e^{i\omega(s_r x_r - t)},$$

where P_k is the polarization vector and $s_r = v^{-1}n_r$ is the slowness vector, with v and n_r being the sound velocity and the wavefront-normal vector, respectively. The sound velocity v can be obtained by solving the eigen-value Christoffel equation,

$$\left(c_{ijkl}n_j n_l - \rho v^2\right)P_k = 0.$$

By using the Voigt notation, the fourth-order stiffness tensor c_{ijkl} can be transformed into a second-order 6 × 6 tensor C_{ij}. For a material with the tetragonal symmetry, C_{ij} has the form of

$$\begin{pmatrix} C_{11} & C_{12} & C_{13} & 0 & 0 & 0 \\ C_{12} & C_{11} & C_{13} & 0 & 0 & 0 \\ C_{13} & C_{13} & C_{33} & 0 & 0 & 0 \\ 0 & 0 & 0 & C_{44} & 0 & 0 \\ 0 & 0 & 0 & 0 & C_{44} & 0 \\ 0 & 0 & 0 & 0 & 0 & \frac{C_{11}-C_{12}}{2} \end{pmatrix}.$$

The Christoffel equation can then be written as

$$\begin{pmatrix} \Gamma_{11}-\rho v^2 & \Gamma_{12} & \Gamma_{13} \\ \Gamma_{12} & \Gamma_{22}-\rho v^2 & \Gamma_{23} \\ \Gamma_{13} & \Gamma_{23} & \Gamma_{33}-\rho v^2 \end{pmatrix}\begin{pmatrix} P_1 \\ P_2 \\ P_3 \end{pmatrix} = 0,$$

where

$$\Gamma_{11} = n_1^2 C_{11} + n_2^2 \frac{C_{11}-C_{12}}{2} + n_3^2 C_{44},$$

$$\Gamma_{22} = n_1^2 \frac{C_{11}-C_{12}}{2} + n_2^2 C_{11} + n_3^2 C_{44},$$

$$\Gamma_{33} = n_1^2 C_{44} + n_2^2 C_{44} + n_3^2 C_{33},$$

$$\Gamma_{23} = n_2 n_3 (C_{13} + C_{44}),$$

$$\Gamma_{13} = n_1 n_3 (C_{13} + C_{44}),$$

$$\Gamma_{12} = n_1 n_2 \frac{C_{11}+C_{12}}{2}.$$

For the acoustic waves propagating along the z axis, the eigen modes from the Christoffel equation should include one longitudinal wave with the velocity $v_l = \sqrt{\frac{C_{33}}{\rho}}$ and two transverse waves with the velocity $v_t = \sqrt{\frac{C_{44}}{\rho}}$, all being polarized along $P_k = (0,0,1)$. If the system has a quasi-cubic symmetry, we would have $C_{33} \cong C_{11}$.

Piezoelectric coupling. Since the phonons could cause the displacement and hence the strain field, we would expect a piezoelectric coupling between the electrons and acoustic phonons in a single $CsPbI_3$ NC. This strain, via the piezoelectric tensor $e_{k;ij}$, is able to create an electric field **E** that interacts with the electron or hole. The electric displacement component D_k (k = x, y or z) in the presence of the piezoelectric effect can be described by

$$D_k = 4\pi e_{k;ij} S_{ij} + \epsilon E_k,$$

where S_{ij} is the strain tensor. The lattice displacement caused by the λth branch of the acoustic phonons can be expressed by

$$u_{i\lambda} = \left(\frac{\hbar}{2\rho V \omega_\lambda(q)}\right)^{1/2} \xi_i(q,\lambda)\left(b_{q\lambda} + b^+_{-q\lambda}\right),$$

where $b^+_{q\lambda}$ creates an acoustic phonon with the eigenvector $\xi_i(q,\lambda)$ in the λth branch at a wave vector q, $\omega_\lambda(q)$ is the phonon dispersion, and V is the volume. Then the piezoelectric coupling H_p can be written as

$$H_p = \sum_{q\lambda} C_\lambda(q) c^+_{k+q}\, c_k \left(b_{q\lambda} + b^+_{-q\lambda}\right),$$

where c^+_k creates an electron with the wave vector k and the coupling strength is

$$C_\lambda(q) = \frac{4\pi e q_k e_{k;ij} \xi_i(\lambda,q) q_j}{\sqrt{V} q^2 \epsilon}\left(\frac{\hbar}{2\rho\omega_\lambda}\right)^{1/2}.$$

If we assume that the $CsPbI_3$ NC is in a tetragonal phase with the C_{4v} symmetry, the nonzero elements of $e_{k;ij}$ are $e_{z;xx} = e_{z;yy} = e_{31}$, $e_{x;zx} = e_{y;zy} = e_{15}$ and $e_{z;zz} = e_{33}$, and the piezoelectric coupling strength reads

$$C_\lambda(q) = \frac{4\pi e}{q^2\epsilon}\left(\frac{\hbar}{2\rho\omega_\lambda}\right)^{1/2} \left[e_{15}\left(q_x^2 + q_y^2\right)\xi_z + e_{33} q_z^2 \xi_z + (e_{15} + e_{31}) q_z \left(q_x \xi_x + q_y \xi_y\right)\right].$$

This piezoelectric coupling strength can be rewritten as

$$C_\lambda(q) = \left(\frac{2\pi g_\lambda}{V}\right)^{1/2} \frac{\hbar v_\lambda}{\sqrt{q}}$$

in term of the dimensionless coupling constant $g_\lambda = \bar{p}_\lambda e^2/(\hbar\epsilon v_\lambda)$, which contains the fine structure constant for the speed of sound $e^2/(\hbar\epsilon v_\lambda)$ and the electro-mechanical coupling $\bar{p}_\lambda$. The orientational average of $\bar{p}_\lambda$,

$$\bar{p}_\lambda = \frac{4\pi}{\epsilon\rho}\langle\left[\frac{e_{k;ij}q_k q_j \xi_i}{q\omega_\lambda}\right]^2\rangle,$$

can be evaluated in the spherical coordinates. The longitudinal electric fields are e_{rrr} for an LA wave propagating in the radial direction and $e_{r\theta r}$ for a TA wave [refs. S2,S3], which satisfy the following expressions,

$$\langle e_{rrr}^2\rangle = \frac{1}{7}e_{33}^2 + \frac{4}{35}e_{33}(e_{31}+2e_{15}) + \frac{8}{105}(e_{31}+2e_{15})^2,$$

$$\langle e_{r\theta r}^2\rangle = \frac{16}{35}e_{15}^2 + \frac{16}{105}e_{15}(e_{33}-e_{31}-e_{15}) + \frac{2}{35}(e_{33}-e_{31}-e_{15})^2.$$

Accordingly, the orientation-averaged electro-mechanical couplings are

$$\bar{p}_L = \frac{8\pi}{35\rho\epsilon c_L^2}\left[\frac{4}{3}\left(e_{31}+\frac{3}{4}e_{33}+2e_{15}\right)^2 + \frac{7}{4}e_{33}^2\right],$$

$$\bar{p}_T = \frac{8\pi}{35\rho\epsilon c_T^2}\left[\left(e_{11}-e_{33}-\frac{1}{3}e_{15}\right)^2 + \frac{56}{9}e_{15}^2\right].$$

Although the piezoelectric parameters are not available for the perovskite $CsPbI_3$, we can use those values obtained previously from the perovskite $MAPbI_3$ to estimate the piezoelectric coupling strengths. In $MAPbI_3$, the dimensionless piezoelectric strengths are disparate for TA and LA phonons, with g_T = 13.8 and g_L = 0.30, respectively. The huge g_T in $MAPbI_3$ is the result of a strong electromechanical coupling due to its ionic feature, and a low speed of sound due to its elastic softness for the transverse modes.

Emission and absorption spectra. We model the emission and absorption spectra of single $CsPbI_3$ NCs by explicitly including the effect of acoustic-phonon scattering. For clarity, we consider a single exciton state that couples to a phonon bath but note that the formalism can be

readily extended to the case with multiple discrete exciton states. The exciton-phonon Hamiltonian can be written as

$$H = E_0|X\rangle\langle X| + \sum_{q\lambda} \hbar\omega_{q\lambda} b_{q\lambda}^{+} b_{q\lambda} + \sum_{q\lambda} \hbar\omega_{q\lambda}\left(f_{q\lambda} b_{q\lambda} + f_{q\lambda}^{*} b_{q\lambda}^{+}\right)|X\rangle\langle X|,$$

where $|X\rangle$ and E_0 are the exciton state and its energy. The second term describes the phonon bath with $b_{q\lambda}^{+}$ ($b_{q\lambda}$) creating (annihilating) a phonon of momentum q in the λth branch, which can be either longitudinal or transverse. The third term is the exciton-phonon interaction characterized by the dimensionless coupling strength $f_{q\lambda}$, which includes both deformational and piezoelectric couplings. Because of the exciton-phonon coupling, both optical absorption to the exciton and emission from the exciton are accompanied by the absorption and emission of acoustic phonons.

For the emission from the exciton, the transition probability W_p that involves p phonons of mode $q\lambda$ can be evaluated according to the independent-boson theory [ref. S4],

$$W_p = \left(\frac{n_{q\lambda}}{n_{q\lambda}+1}\right)^{p/2} e^{-|f_{q\lambda}|^2(2n_{q\lambda}+1)} I_p\left(2|f_{q\lambda}|^2\sqrt{n_{q\lambda}(n_{q\lambda}+1)}\right),$$

where I_p is the modified Bessel function of order p and $n_{q\lambda} = \frac{1}{e^{-\hbar\omega_{q\lambda}/k_B T}-1}$ is the equilibrium phonon occupation at the temperature of T.

Each value of p would contribute a discrete line with height W_p around the zero-phonon line (ZPL). A positive (negative) p means that $|p|$ phonons of mode $q\lambda$ are absorbed (emitted) during the optical transition. The total emission spectrum can be calculated according to

$$I_e(\omega) = \sum_{p_1,\ldots,p_N} W_{P_1} W_{P_2} \ldots W_{P_N} \delta\left(\hbar\omega - E_0 - \sum_i p_i \hbar\omega_i\right),$$

where i represents phonon mode $(q\lambda)$ and the summation over phonon number p_i is from $-\infty$ to $+\infty$. The δ-function of energy conservation can be replaced by a Lorentzian function

$$\delta(\hbar\omega - E_0 - \sum_i p_i \hbar\omega_i) \approx \frac{1}{\pi} \frac{\Gamma}{(\hbar\omega - E_0 - \sum_i p_i \hbar\omega_i)^2 + \Gamma^2},$$

where Γ is the exciton broadening. The optical absorption spectrum can be similarly expressed as

$$I_a(\omega) = \sum_{p_1,\ldots,p_N} W'_{P_1} W'_{P_2} \ldots W'_{P_N} \delta\left(\hbar\omega - E_0 - \sum_i p_i \hbar\omega_i\right),$$

$$W'_p = \left(\frac{n_{q\lambda}}{n_{q\lambda}+1}\right)^{-p/2} e^{-|f_{q\lambda}|^2 (2n_{q\lambda}+1)} I_p\left(2|f_{q\lambda}|^2 \sqrt{n_{q\lambda}(n_{q\lambda}+1)}\right).$$

Since both W_p and W'_p decrease with $|p|$ rapidly, the emission and absorption spectra can be adequately simulated by considering only $|p| < 3$.

In Supplementary Fig. 7, we display the PL intensity as a function of the detuned energy $\hbar\omega - E_0$ at T = 3 K, which is calculated for the PL spectrum shown in Fig. 4a of the main text from a single $CsPbI_3$ NC under resonant excitation of its higher-energy peak. We consider a strong piezoelectric coupling with TA phonons ($\hbar\omega_q =$ 155 μeV) and a relatively weak piezoelectric/deformational coupling with LA phonons ($\hbar\omega_q =$ 505 μeV), with their dimensionless coupling strengths being assumed to be $|f|$ = 0.06 and 0.02, respectively. The exciton broadening Γ is set to be 17.6 μeV that is consistent with the experimental result. We see that the one-phonon peak of 155 μeV emerges both above and below the ZPL. The peak below the ZPL, which corresponds to the phonon emission, has a higher intensity because the emission (absorption) of phonon is proportional to $n_{q\lambda} + 1$ ($n_{q\lambda}$) and the difference can be significant at low temperatures when $n_{q\lambda} \ll 1$. This also explains that a pronounced one-phonon peak of 505 μeV appears only below the ZPL, which, however, is not resolvable from

the background due to the limited signal-to-noise ratio in our experiment. Two and higher phonon peaks for both 155 μeV and 505 μeV are too weak to be clearly distinguished from the natural broadening. It is remarkable that our model with only two phonon modes can account for the measured PL spectrum very satisfactorily.

The calculated absorption spectra for a single $CsPbI_3$ NC are plotted in Supplementary Fig. 6, which are related to the PL excitation spectra shown in Fig. 3b of the main text. The measured PL excitation spectra at 3 and 10 K can be well accounted for by considering the two TA (190 μeV) and LA (626 μeV) phonon modes with the dimensionless coupling strengths of $|f| = 0.06$ and 0.02, respectively. It is interesting to note that in the absorption spectrum, it is the phonon peaks above the ZPL that are more pronounced. At $T = 3$ K, the exciton broadening Γ is set to be 17.6 μeV and it becomes 64 μeV at $T = 10$ K. The larger broadening at $T = 10$ K makes the discrete phonon peaks less pronounced.

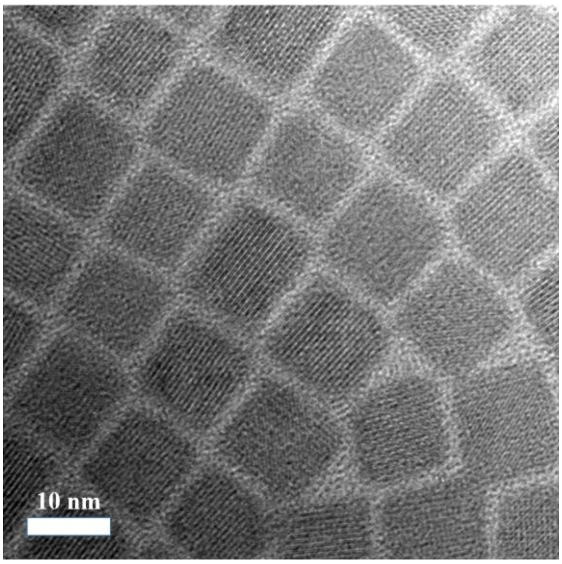

Supplementary Fig. 1. Transmission electron microscope image measured for perovskite $CsPbI_3$ NCs.

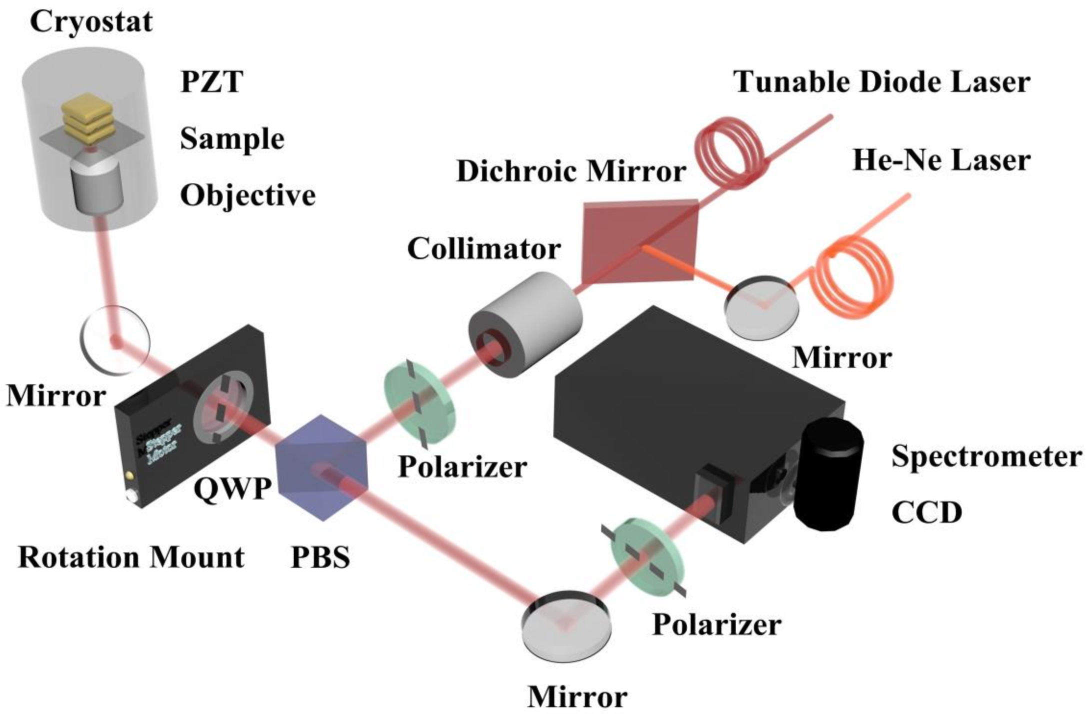

Supplementary Fig. 2. Experimental setup for the optical studies of single $CsPbI_3$ NCs. PZT: piezo-electric transducer; QWP: quarter-wave plate; PBS: polarization beam splitter; CCD: charge-coupled device. The laser beam first passes through a linear polarizer and is then reflected by a PBS, which are respectively chosen to transmit and reflect light with the vertical linear polarization. After being focused by the objective and scattered by the sample substrate, the laser light collected by the same objective is blocked first by the PBS and then by a horizontal linear polarizer placed before the spectrometer and the CCD camera. To minimize the residual laser light that still arrives at the CCD camera, a QWP is inserted between the PBS and the objective to correct the birefringence effect caused by relevant components in the optical path. Under resonant excitation of the tunable diode laser, a single $CsPbI_3$ NC is always chosen to emit doublet peaks with comparable PL intensities, implying that the two transition dipole moments are aligned at ~45º relative to the laser polarization direction. In this case, part of the doublet-peak photons emitted by a single $CsPbI_3$ NC can still arrive at the CCD camera for the resonance/near-resonance fluorescence measurement, after passing through the PBS and the horizontal linear polarizer sequentially.

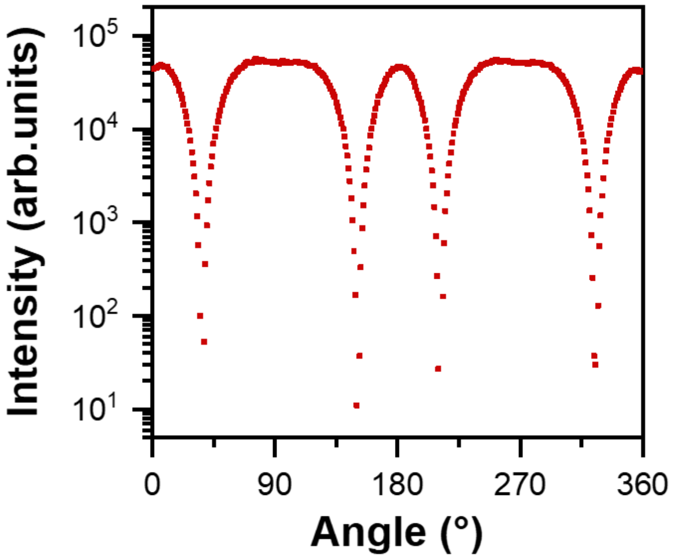

Supplementary Fig. 3. Residual laser light measured on the CCD camera as a function of the rotating angle of the quarter-wave plate.

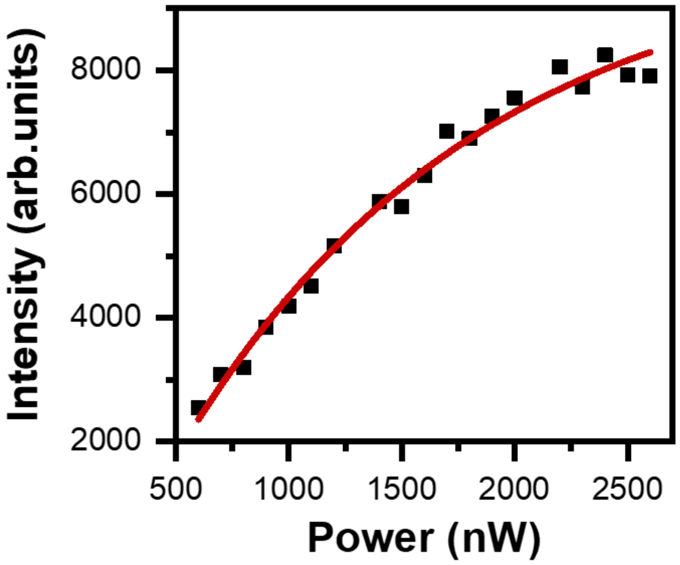

Supplementary Fig. 4. PL intensity measured for a single $CsPbI_3$ NC as a function of the 1.96 eV laser excitation power. The data points are fitted by the solid line using a function form of $I \propto 1 - e^{-\alpha P}$, where I is the PL intensity, α is a fitting constant, and P is the laser power.

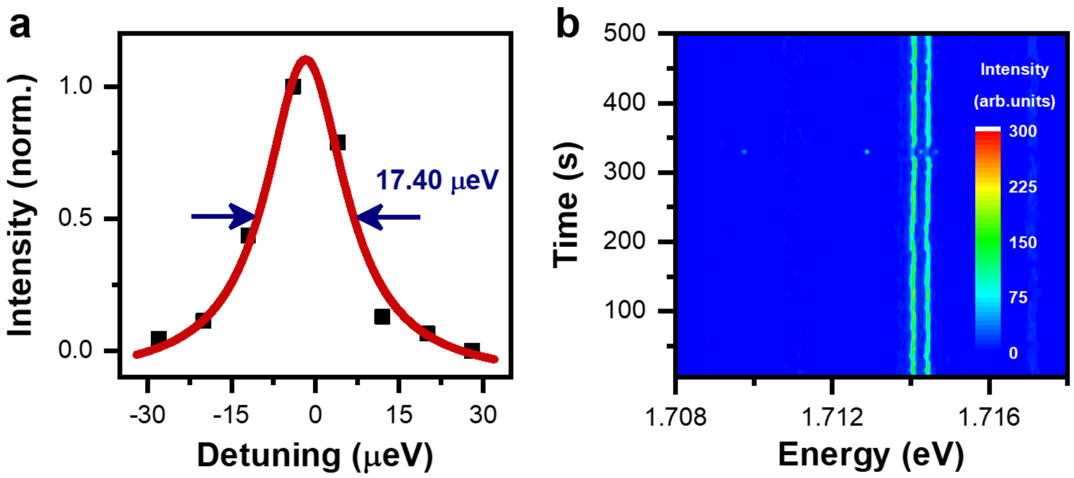

Supplementary Fig. 5. a, PL intensity of the lower-energy peak plotted as a function of the detuned laser energy and fitted with a linewidth of ~17.40 μeV for a single $CsPbI_3$ NC. **b,** Time-dependent PL spectral image constructed from 100 PL spectra each acquired with an integration time of 5 s for this single $CsPbI_3$ NC excited at 1.96 eV.

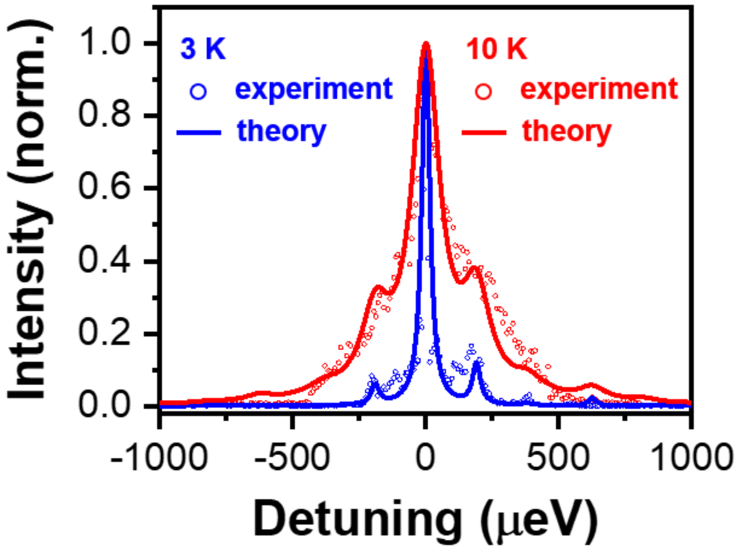

Supplementary Fig. 6. Experimentally measured and theoretically calculated PL excitation spectra for the lower-energy peak of a single $CsPbI_3$ NC at 3 and 10 K, respectively.

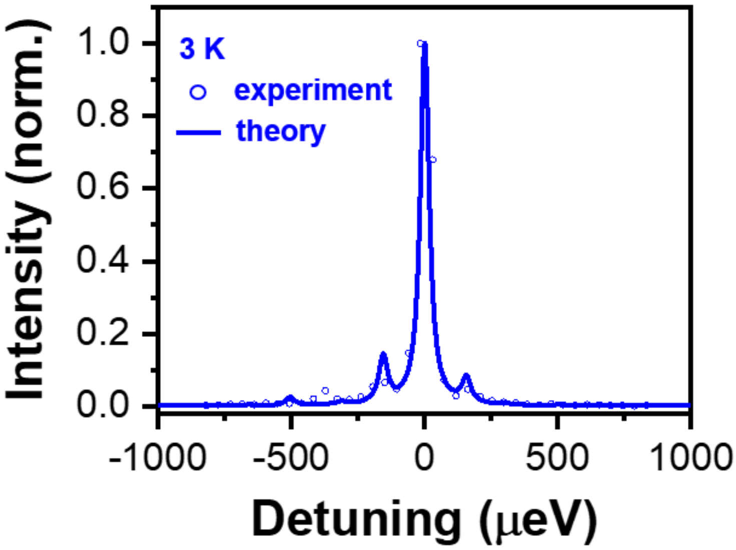

Supplementary Fig. 7. Experimentally measured and theoretically calculated PL spectra for a single $CsPbI_3$ NC under resonant excitation of the higher-energy peak at 3 K. The lower-energy peak located at about -370 μeV is not included in the theoretical fitting.

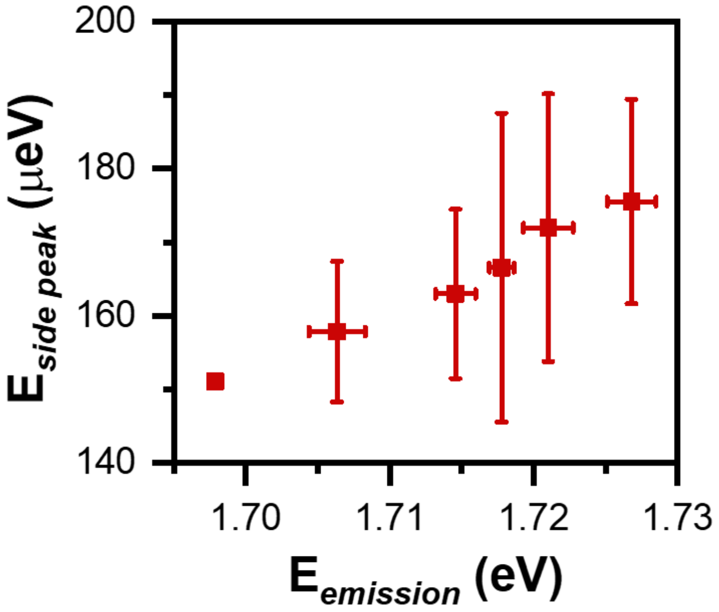

Supplementary Fig. 8. Size-quantized acoustic-phonon energies plotted as a function of the emission energies for a total of 24 single $CsPbI_3$ NCs. The horizontal (vertical) error bar denotes the distribution of emission (acoustic-phonon) energies around each data point.

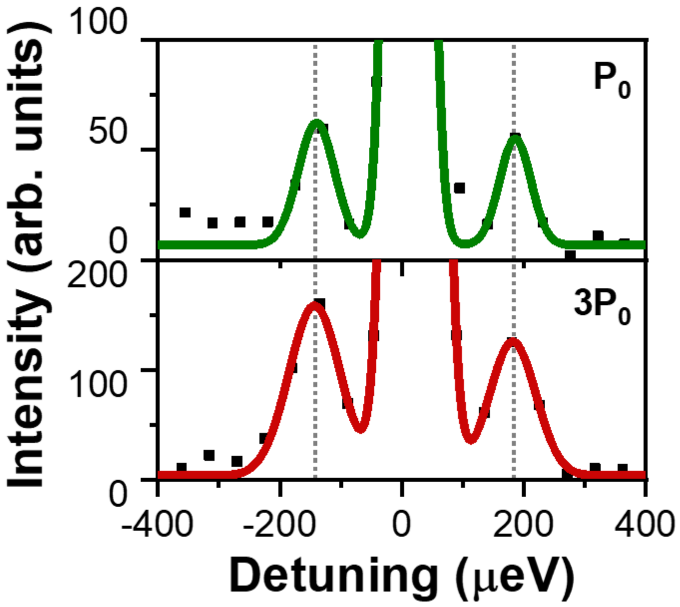

Supplementary Fig. 9. Resonantly-excited PL spectra of a single $CsPbI_3$ NC measured at two different laser powers of P_0 and $3P_0$, showing that the energy separation between the two side peaks is not changed.

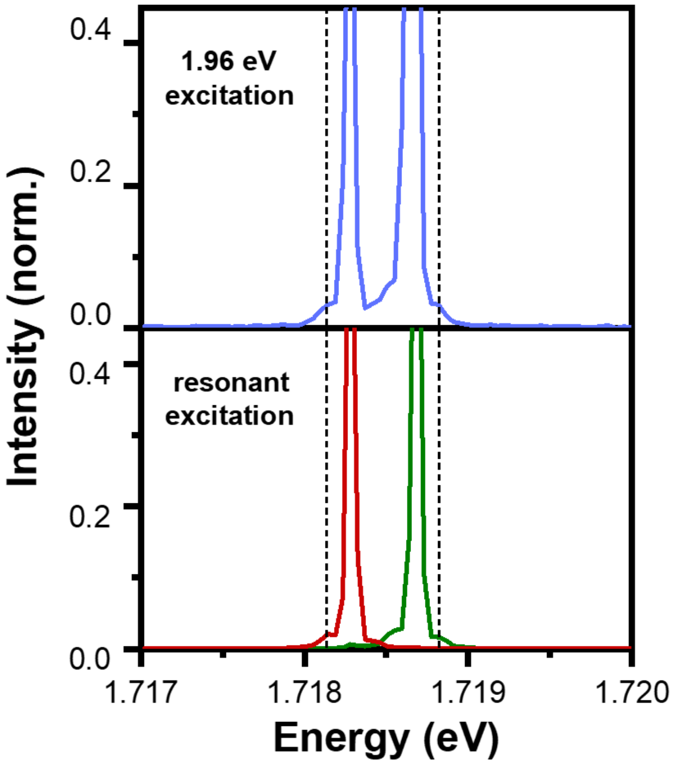

Supplementary Fig. 10. (top panel) PL spectrum of a single $CsPbI_3$ NC excited at 1.96 eV. (bottom panel) Resonantly-excited PL spectra measured for the same single $CsPbI_3$ NC. The energy positions of a size-quantized acoustic-phonon mode are marked by the dotted lines, which are separated from their respective central PL peaks by an energy of ~190 μeV. The top and bottom PL spectra shown here correspond to amplified views of the PL spectra plotted in the top and bottom panels, respectively, in Fig. 1b of the main text for the same single $CsPbI_3$ NC.

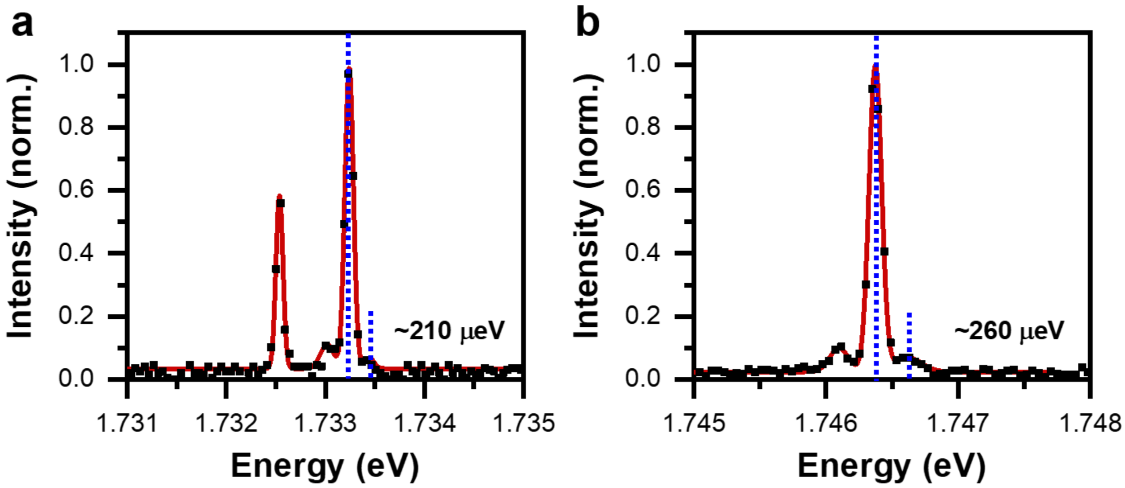

Supplementary Fig. 11. a, PL spectrum of a single $CsPbI_3$ NC excited at 1.96 eV above the bandgap. The higher-energy one of the doublet PL peaks is located at ~1.7332 eV, and the vibration energy of the size-quantized acoustic-phonon mode around it is estimated to be ~210 μeV. **b,** PL spectrum of a single $CsPbI_3$ NC exited at 1.96 eV above the bandgap, where only one PL peak is detected due to the degenerate exciton fine structures within the system resolution of ~100 μeV. The vibration energy of the size-quantized acoustic-phonon mode is estimated to be ~260 μeV, as compared to the central PL peak with an emission energy of ~1.7464 eV.

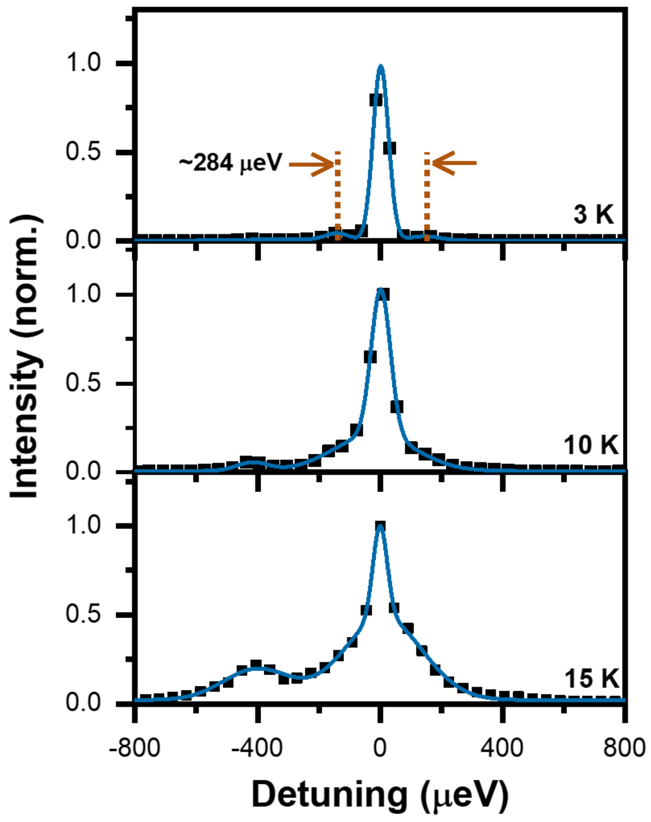

Supplementary Fig. 12. PL spectra measured at 3, 10 and 15 K for a single $CsPbI_3$ NC with the laser excitation energy being resonant with its higher-energy peak at 0 μeV. The size-quantized acoustic-phonon peak and the lower-energy peak of this single $CsPbI_3$ NC are separated from the higher-energy peak by ~142 and ~400 μeV, respectively.
